# Development of a core outcome set for clinical trials aimed at improving antimicrobial stewardship in care homes

**DOI:** 10.1186/s13756-021-00925-8

**Published:** 2021-03-09

**Authors:** Hoa Q. Nguyen, Declan T. Bradley, Michael M. Tunney, Carmel M. Hughes

**Affiliations:** 1 School of Pharmacy, Queen’s University Belfast, 97 Lisburn Road, Belfast, BT9 7BL UK; 2 Faculty of Pharmacy, University of Medicine and Pharmacy At HCMC, 41-43 Dinh Tien Hoang Street, Ben Nghe Ward, District 1, Ho Chi Minh City, Vietnam; 3 Centre for Public Health, School of Medicine, Dentistry and Biomedical Sciences, Queen’s University Belfast, Royal Victoria Hospital, Grosvenor Road, Belfast, BT12 6BA UK; 4 Public Health Agency, 12-22 Linenhall Street, Belfast, BT2 8BS UK

**Keywords:** Core outcome set, Antimicrobial stewardship, Care homes, Outcome measurement instrument

## Abstract

**Background:**

Diverse outcomes reported in clinical trials of antimicrobial stewardship (AMS) interventions in care homes have hindered evidence synthesis. Our main objective was to develop a core outcome set (COS) for use in trials aimed at improving AMS in care homes.

**Methods:**

A refined inventory of outcomes for AMS interventions in care homes, compiled from a previous study, was rated in a three-round international Delphi survey with 82 participants, using a nine-point Likert scale (from 1, unimportant, to 9, critical). This was followed by an online consensus exercise with 12 participants from Northern Ireland to finalise the COS content. Subsequently, a suitable outcome measurement instrument (OMI) was selected for each outcome in the COS by: identifying existing OMIs through a literature search and experts’ suggestions, assessing the quality of OMIs, and selecting one OMI for each core outcome via a two-round international Delphi survey with 59 participants.

**Results:**

Of 14 outcomes initially presented, consensus was reached for inclusion of five outcomes in the COS after the three-round Delphi survey and the online consensus exercise, comprising the total number of antimicrobial courses prescribed, appropriateness of antimicrobial prescribing, days of therapy per 1000 resident-days, rate of antimicrobial resistance, and mortality related to infection. Of 17 potential OMIs identified, three were selected for the two-round Delphi exercise after the quality assessment. Consensus was reached for selection of two OMIs for the COS.

**Conclusion:**

This COS is recommended to be used in clinical trials aimed at improving AMS in care homes.

**Supplementary Information:**

The online version contains supplementary material available at 10.1186/s13756-021-00925-8.

## Background

High rates of antimicrobial prescribing, including inappropriate prescribing, have been reported in care homes [1, 2]. This issue may increase the risk of adverse drug events and antimicrobial resistance (AMR) amongst care home residents [2, 3]. There has been a call to implement antimicrobial stewardship (AMS), a general programme to enhance appropriateness of prescribing and reduce AMR, in this setting [4]. Interventions to improve AMS in care homes have been reported; however, the overall effect was modest [5, 6]. Previous studies indicated that heterogeneity in reported outcomes across trials hindered data synthesis, and several outcomes potentially useful for AMS interventions had not been used in these trials [6, 7].

The Core Outcome Measures in Effectiveness Trials (COMET) initiative has facilitated development of core outcome sets (COSs) to improve the quality and quantity of measured outcomes in research [8]. A COS is defined as a set of important outcomes which are agreed by consensus and should be measured to evaluate the effectiveness of interventions in trials of a specific health area [9]. The COMET initiative also encourages the establishment of how to measure outcomes in a COS to enhance dissemination and implementation [8]. In line with this recommendation, the Consensus-based Standards for the selection of health Measurement Instruments (COSMIN) initiative has published joint guidelines for selection of outcome measurement instruments (OMIs), tools which measure outcomes in terms of quality or quantity, to be used in a COS [10].

To date, no COS has been developed for AMS studies in care homes. The aim of this study was to develop a COS and identify appropriate OMIs that could be used in clinical trials aimed at improving AMS in care homes.

## Methods

**Fig. 1:**
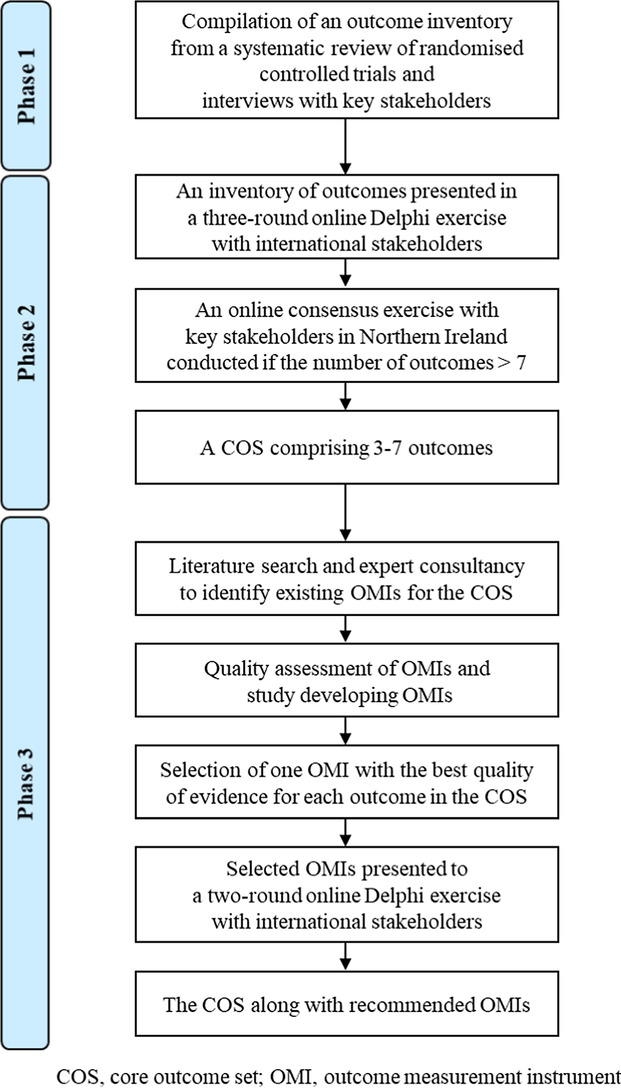
Flow chart outlined the development of a core outcome set along with measurement instruments

The development of the COS followed the COMET and COSMIN guidelines [8, 10]. The study involved three phases: compiling an inventory of potential outcomes, producing a COS through a series of consensus procedures, and selecting OMIs for the COS (Fig. 1). A project steering group (PSG) comprised all members of the research team (HN, DB, MT, and CH).

### Outcome inventory compilation

In Phase 1, an inventory of 14 outcomes was compiled from a previous systematic review of randomised controlled trials and a qualitative study with key stakeholders [6, 7]. These outcomes were grouped into key categories as suggested by the COMET handbook [8], and were included in a subsequent series of consensus exercises (see Additional file 1: Table S1).

### Consensus procedures to develop the COS

Phase 2 involved a three-round online Delphi survey and an online consensus exercise with relevant stakeholders to reach consensus on the COS comprising up to seven outcomes. Ethical approval was granted by the School of Pharmacy Research Ethics Committee at Queen’s University Belfast (Reference number: 014PMY2019).

#### Outcome Delphi consensus survey (10/2019–12/2019)

An electronic questionnaire for the Delphi exercise was developed using a web-based survey tool, comprising the inventory of outcomes along with plain English definitions. Participants were asked to assign a score between 1 and 9 according to how they judged the importance of each outcome (1–3, unimportant; 4–6 important but not critical; 7–9, critical). The participants were also asked to suggest any additional outcomes at the end of the first round. The questionnaires were piloted with other researchers at the School of Pharmacy, Queen’s University Belfast and modified according to feedback.

The Delphi panel comprised researchers, healthcare professionals who had experience of AMS or providing care in care homes, and members of advocacy groups for older people. Care home residents were not recruited due to concerns that some may lack capacity and ability to participate. Participants were also asked to suggest other potential stakeholders for the study.

The Delphi survey comprised three sequential rounds, with group and individual feedback on responses from previous rounds provided to participants in Round 2 and 3. Consenting participants were emailed a link to access the online questionnaire. Reminder emails were also sent to facilitate completion. Only participants who had completed a round were invited for the next round. Round 2 comprised all outcomes in Round 1 and additional outcomes suggested by the Delphi panel. Round 3 comprised outcomes for which no consensus had been reached in Round 2.

#### Online consensus exercise (04/2020–05/2020)

A face-to-face meeting, using the Nominal Group Technique (NGT) [11], had been planned to finalise the COS if the number of outcomes included after the Delphi survey was more than seven. For logistical reasons, only key stakeholders from Northern Ireland (NI) were invited. However, the scheduled meeting was cancelled due to the COVID-19 pandemic. Therefore, an online consensus exercise with two online questionnaires, based on the NGT approach, was undertaken. In the first questionnaire, each consenting participant, including the PSG members, was asked to select up to seven outcomes important to them, along with a brief explanation. A report on this questionnaire was provided to the participants in the second questionnaire, who were then asked to score each outcome between 1 and 9 according to how they judged the importance, similarly to the questionnaires in the previous Delphi exercise.

### Selection of OMIs for the COS

After the COS was defined through consensus procedures, Phase 3 was undertaken to select OMIs for the COS, following the COSMIN guidelines [10]. Ethical approval for this phase was granted by the Faculty Research Ethics Committee at Queen’s University Belfast (Reference number: MHLS 20_50).

#### Finding existing OMIs

A literature search was undertaken to identify studies reporting OMIs for each outcome in the COS (up to February 29th, 2020), using Medline, Embase, the COSMIN database and four grey literature resources (see see Additional file 1: Table S2). Reference lists of identified studies and previous systematic reviews of AMS in care homes [5, 6, 12] were also screened for potential studies and OMIs. Non-English publications were excluded. Subsequently, an inventory of identified OMIs was reviewed with four experts, who had conducted research on antimicrobial prescribing in care homes.

#### Quality assessment of OMIs

The methodological quality of studies developing OMIs, identified through the literature search, was assessed using the adapted COSMIN checklist [13–15]. Each OMI identified in the literature search was classified into ‘objective’ (i.e. independent from individual judgment) or ‘subjective’ (i.e. potentially having different results based on different assessors’ judgments) through discussion with all PSG members. Subsequently, all OMIs were assessed for content validity and feasibility aspects, and ‘subjective’ OMIs were also assessed for other measurement properties (if applicable) [10]. Two researchers (HN and CH/MT) independently assessed the quality of OMIs, and any inconsistency was discussed with another researcher (MT, CH or DB). Criteria for good measurement properties described by the COSMIN guidelines were applied for each OMI [10].

Following the quality assessment, the PSG reviewed and selected one OMI, which had the best overall quality of evidence and at least high quality of evidence for good content validity, for each outcome in the COS to be assessed in a subsequent consensus procedure. An outcome for which all OMIs did not meet this minimum requirement was reported with the comment ‘OMI not available’.

#### OMI Delphi consensus survey (07/2020–08/2020)

A two-round online Delphi technique with international stakeholders was undertaken to reach consensus on selection of OMIs. The PSG developed an electronic questionnaire which included a list of OMIs selected for the COS, along with their definitions and illustrative examples. This questionnaire, embedded in a web-based tool, was piloted with other researchers at the School of Pharmacy before distribution.

Participants who had taken part in the previous Delphi survey were invited. Snowball sampling was also applied to enhance further recruitment. In the first questionnaire, consenting participants were asked whether they agreed the OMIs presented should be used for the COS, and to provide a brief explanation. Only participants who had completed Round 1 were invited to participate in Round 2. In this round, participants were informed of their previous response, a summary of the group responses, along with brief responses of the PSG to comments from the Delphi panel. Participants were asked to rate the OMIs again without explaining their rationale.

### Data analysis

All statistical analysis was performed using R software version 4.0.2. Consensus criteria, based on previous studies [8], were set a priori. In Round 2 and 3 of the outcome Delphi survey, any outcome with a rating of 7 to 9 by 80% or more of participants and 1 to 3 by 15% or fewer was included in the COS; any outcome with a rating of 7 to 9 by 15% or more of participants and 1 to 3 by 80% or fewer was excluded; any outcome with other scores were considered as ‘no consensus’ which were retained after Round 2 or excluded after Round 3. During the online consensus exercise, consensus was reached for inclusion of an outcome in the COS when 80% or more of participants scored between 7 and 9; if the total number of included outcomes was less than three, outcomes with a rating 7 to 9 by 70% or more were also included as ‘optional outcomes’. Regarding the second Delphi survey for selection of OMIs for the COS, consensus was reached for inclusion of an OMI when 70% or more of participants agreed, and 15% or fewer disagreed; OMIs with other scores were excluded.

## Results

### Outcome inventory compilation

The inventory of 14 outcomes were classified into four categories: delivery of care (eight outcomes), infection outcome (three outcomes), hospitalisation (one outcome), and mortality/survival (two outcomes) (see Additional file 1: Table S1).

### Consensus procedures to develop the COS

#### Outcome Delphi consensus survey (three rounds)

Of 239 potential participants invited, 86 (36%) provided informed consent and were enrolled in the Delphi exercise. The first questionnaire was completed by 82 participants from 17 countries (response rate: 95.3%) The demographics of the Delphi panel are detailed in Table 1. The summary of rating of Delphi panel members is presented in Table S3 (see Additional file 1).

**Table 1:** Demographic profile of participants in the outcome Delphi survey

Characteristic	Round 1	Round 2	Round 3
Participant, n	82	77	75
**Gender, n (%)**			
Female	52 (63.4)	49 (63.6)	48 (64.0)
Male	30 (36.6)	28 (36.4)	27 (36.0)
**Continent of residence, n (%)**			
Europe	48 (58.5)	44 (57.1)	42 (57.3)
America	21 (25.6)	21 (27.3)	21 (28.0)
Asia	10 (12.2)	9 (11.7)	7 (10.7)
Australia	3 (3.7)	3 (3.9)	3 (4.0)
**Professional area, n (%)**^a^			
Pharmacist	35 (42.7)	34 (42.9)	33 (44.0)
Doctor	28 (34.1)	27 (33.8)	26 (34.7)
Researcher	26 (31.7)	25 (32.5)	25 (33.3)
Nurse	6 (7.3)	6 (7.8)	6 (8.0)
Care home staff	1 (1.2)	1 (1.3)	1 (1.3)
Representative of advocacy groups	4 (4.9)	2 (2.6)	2 (2.7)
Other^b^	2 (2.4)	2 (2.6)	2 (2.7)
Age, median (range)	45 (23—74)	46 (29—74)	46 (29—74)

^a^Percentages do not add to 100% because some participants selected more than one professional area

^b^Includes: microbiologist, health economist

**Fig. 2:**
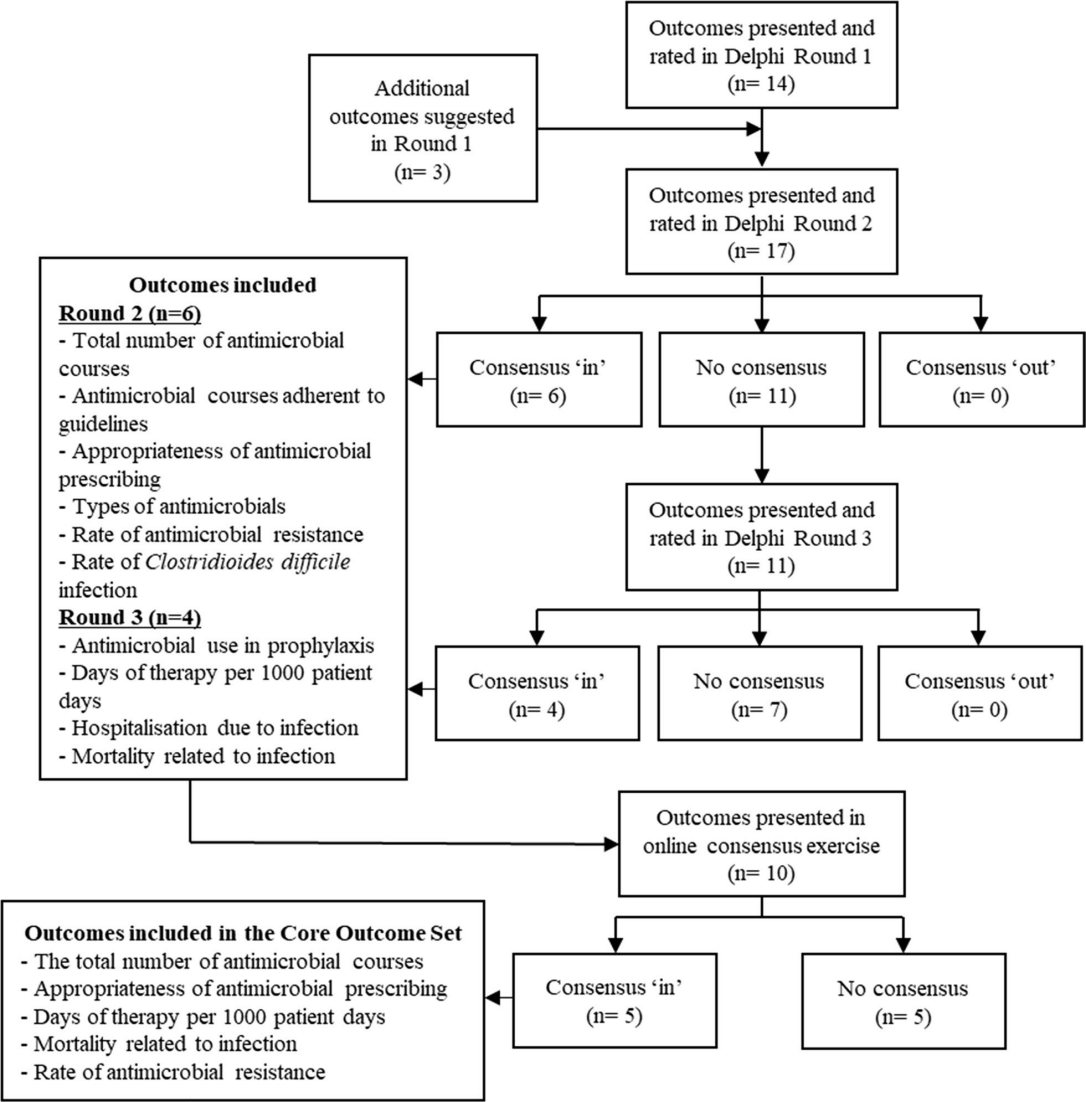
Consensus procedures and the final core outcome set

After Round 1, comments from the Delphi panel were reviewed and three new outcomes (‘Proportion of broad- and narrow-spectrum antimicrobials’, ‘Use of laboratory tests’, and ‘Emergency department visits’) were added to the second-round questionnaire. In Round 2, 77 participants completed the questionnaire (response rate: 93.9%). Consensus was reached for inclusion of six outcomes. Eleven outcomes for which consensus was not reached were then presented in Round 3. Seventy-five participants completed the third questionnaire (response rate: 97.4%), and consensus was reached for inclusion of four more outcomes (Fig. 2).

#### Online consensus exercise

As the number of outcomes included after the Delphi exercise was greater than seven, the online consensus exercise was undertaken with twelve people from NI (median age: 47.5 years; five females), including four PSG members. Apart from the PSG, other participants were two doctors, two pharmacists, one microbiologist, one researcher, one care home manager and one representative of older people.

All twelve participants completed the first online questionnaire, and eleven completed the second questionnaire (response rate: 91.7%). The summary of ratings in the online consensus exercise is presented in Table S4 (see Additional file 1). Consensus was reached for inclusion of two main outcomes (‘The total number of antimicrobial courses prescribed’ and ‘Appropriateness of antimicrobial prescribing’) and three optional outcomes (‘Days of therapy per 1000 resident-days’, ‘Mortality related to infection’ and ‘Rate of antimicrobial resistance’) in the COS (Fig. 2).

### Selection of OMIs for the COS

#### Finding existing OMIs

**Fig. 3:**
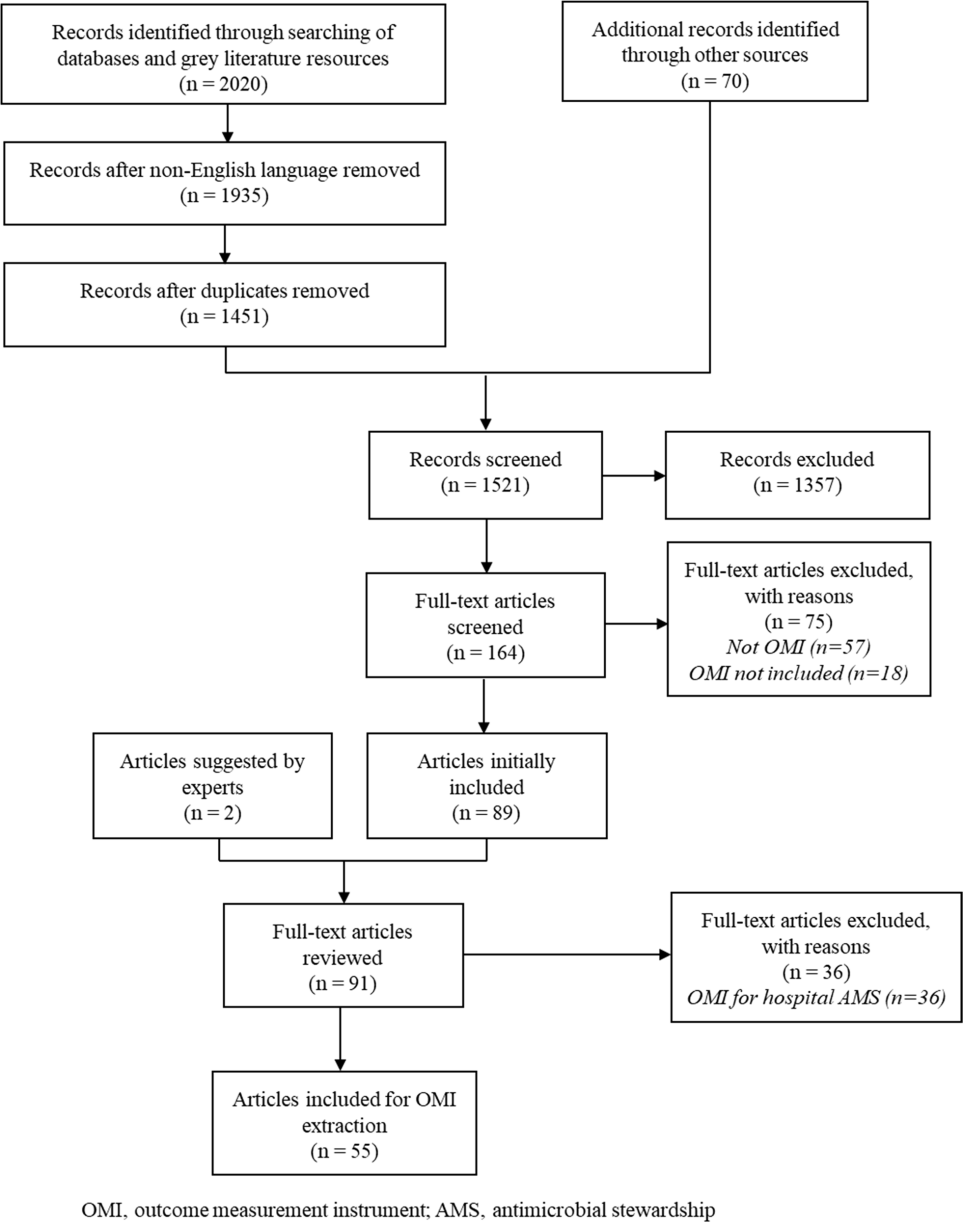
Process of screening and selecting articles to extract relevant outcome measurement instruments

A total of 2020 records were retrieved from the literature search. After duplicate removal and article screening, 89 articles were initially included to compile an initial inventory of OMIs which was then discussed with four experts (from Scotland, Ireland, the United States and Canada). No additional OMIs were recorded but two additional articles were suggested. These 91 articles were reviewed to select those which reported OMIs for the COS. Finally, 55 articles, including ten studies developing OMIs, were selected (see Additional file 1: Table S5). Based on these 55 articles, a list of 17 OMIs was compiled (Table 2). The detailed process of selecting articles is presented in Fig. 3.

**Table 2:** Quality assessment of outcome measurement instruments

Outcome	Outcome measurement instrument	Objective/Subjective	Overall quality of evidence
The total number of antimicrobial courses prescribed	(1) Number of antimicrobial courses started per 1000 resident-days [16–18]	Objective	+
	(2) Number of antibiotic transactions per 1000 resident-days [19]	Objective	?
	(3) Point prevalence of antimicrobial use [20]	Objective	?
	(4) Total number of antimicrobial courses [21]	Objective	?
	(5) Mean number of residents treated with antimicrobial per month [22]	Objective	?
Appropriateness of antimicrobial prescribing	(6) Loeb minimum criteria for initiating antibiotic therapy in SSTIs, RTIs, UTIs, fever where the focus of infection is unknown [23]	Subjective	?
	(7) Revisited McGeer criteria for diagnosing infection: SSTIs, RTIs, UTIs, gastrointestinal tract infections, systemic infections [24, 25]	Subjective	-
	(8) Crnich algorithm for the initiation of antibiotics for UTIs [26]	Subjective	-
	(9) Van Buul algorithms to evaluate appropriateness of initiating or withholding antibiotics in SSTIs, RTIs, UTIs [27]	Subjective	?
	(10) The Medication Appropriateness Index [28, 29]	Subjective	-
Days of therapy per 1000 resident-days	(11) Rate of antimicrobial days of therapy per 1000 resident-days [16–18]	Objective	+
	(12) Antibiotic utilization ratio [16–18]	Objective	?
Rate of antimicrobial resistance	(13) Number of cases with specific (non-) resistant organisms [30]	Subjective	-
	(14) Number of specific (non-) resistant isolates/ organisms [31, 32]	Subjective	-
	(15) Drug Resistance Index [33]	Subjective	-
Mortality related to infection	(16) Rate of mortality related to infection per 1000 resident-days [34]	Subjective	-
	(17) Proportion of mortality related to infection [30]	Subjective	-

+, positive rating; ?, indeterminate rating; −, negative rating

RTIs, respiratory tract infections; SSTIs, skin and soft tissue infections; UTIs, urinary tract infections

#### Quality assessment of OMIs

The methodological quality of ten studies developing OMIs is presented in Table S6 (see Additional file 1). The summary of quality assessment of 17 OMIs compiled from 55 identified articles is presented in Table 2 and Tables S7-9 (see Additional file 1).

Based on the findings of the quality assessment, the OMIs ‘Number of antimicrobial courses started per 1000 resident-days’, ‘Rate of antimicrobial days of therapy per 1000 resident-days’, and ‘Van Buul algorithms’ were selected for a subsequent consensus procedure. No OMIs were selected for the outcomes ‘Rate of antimicrobial resistance’ and ‘Mortality related to infection’ as their OMIs did not meet the minimum requirement for selection.

#### OMI Delphi consensus survey (two rounds)

The three selected OMIs along with their definitions and illustrative examples were presented in the second Delphi consensus exercise. Of 121 stakeholders approached, 68 (56.2%) accepted the invitation, and 59 (from 16 countries) provided informed consent and completed the first questionnaire. The demographic details of the OMI Delphi panel are summarised in Table 3. The summary of agreement levels for the three OMIs is presented in Table S10 (see Additional file 1).

**Table 3:** Demographic profile of participants in the OMI Delphi panel

Characteristic	Round 1	Round 2
Participant, n	59	54
**Gender, n (%)**		
Female	41 (69.5)	38 (70.4)
Male	18 (30.5)	16 (29.6)
**Continent of residence, n (%)**		
Europe	32 (54.2)	30 (55.6)
America	16 (27.1)	15 (27.8)
Asia	6 (10.2)	6 (11.1)
Australia	5 (8.5)	3 (5.5)
**Professional area, n (%)**^a^		
Pharmacist	27 (45.8)	26 (48.1)
Doctor	19 (32.2)	17 (31.5)
Researcher	19 (32.2)	16 (30.8)
Nurse	4 (6.8)	3 (5.5)
Care home manager/staff	1 (1.7)	1 (1.9)
Representative of advocacy groups	1 (1.7)	1 (1.9)
Other^b^	3 (5.1)	3 (5.8)
Age, median (range)	44 (24–75)	43.5 (24–75)

^a^Percentages do not add to 100% because some participants selected more than one professional area

^b^Includes: one microbiologist, two higher education lecturers

In Round 1, the questionnaire was completed by 59/68 participants (response rate: 86.8%). In Round 2, 54 participants completed the questionnaire (response rate: 91.5%). Consensus was reached to select two OMIs ‘Number of antimicrobial courses started per 1000 resident-days’ and ‘Rate of antimicrobial days of therapy per 1000 resident-days’. Consensus was not reached to select the OMI ‘Van Buul algorithms’. The final COS, along with selected OMIs, is presented in Table 4.

**Table 4:** The COS use in trials aimed at improving AMS in care homes and recommended OMIs

Outcome	Outcome definition	Recommended OMI
**Delivery of care**		
The total number of antimicrobial courses prescribed (Main outcome)	The total number of antimicrobial courses that are prescribed for care home residents over a period of time (e.g. over a month, or a year)	This outcome should be measured by the OMI ‘Incidence of antimicrobial use’. This OMI is defined as the number of antibiotic courses started per 1000 resident-days. This can be calculated by the following formula: (Number of antimicrobial courses started / number of resident-days) × 1000 + An antimicrobial course started is defined as all antimicrobials given continuously for one particular indication. This is deemed to be one course (including antimicrobial switch or extension of treatment duration). Antimicrobials prescribed for a second indication during the same period or prescribed after an antimicrobial-free duration of seven days for the same indication is deemed to be a separate course. In the case that treatment documentation cannot be identified, prescription date and treatment duration can be used to deduce an antimicrobial course. An antimicrobial-free duration of seven days can be applied to identify a new course + A resident day is defined as each date of service in which a care home resident was present in the facility and received services
Days of therapy per 1000 resident-days (Optional outcome)	The duration (in days) of antimicrobial courses that are prescribed for care home residents, standardised to 1000 resident-days	This outcome should be measured by the OMI ‘Rate of antimicrobial days of therapy per 1000 resident-days’. This OMI can be calculated by the following formula: (Number of antimicrobial days of therapy / Number of resident-days) × 1000 + An antimicrobial day of therapy is defined as each day a care home resident is administered a systemic antimicrobial agent + A resident day is defined as each date of service in which a care home resident was present in the facility and received services + It is noted that this OMI should be applied separately for antimicrobials for TREATMENT of infection and PREVENTION of infection (infection prophylaxis)
Appropriateness of antimicrobial prescribing (Main outcome)	Antimicrobial courses that are prescribed in accordance with the best available evidence and are suitable for a patient, taking their medical history and medical conditions (including infection) into consideration	OMI not available
**Mortality/survival**		
Mortality related to infection (Optional outcome)	The number of deaths of care home residents related to infection	OMI not available
*Infection*		
Rate of antimicrobial resistance (Optional outcome)	The number of cases in which antimicrobial-resistant bacteria are identified	OMI not available

*OMI* outcome measurement instrument

## Discussion

This study followed the COMET and COSMIN guidelines to develop a COS along with OMIs for use in clinical trials aimed at improving AMS in care homes. Consensus was reached for inclusion of five outcomes and two OMIs in the COS. The adoption of this COS in future studies may enhance interpretation and evidence synthesis of AMS interventions in care homes.

Although no OMIs were selected for three outcomes in the COS, available literature has suggested potential approaches for measurement. Future trials may gauge appropriateness of antimicrobial prescribing by using practice guidelines as a surrogate outcome. Adherence to antimicrobial guidelines has been measured in a number of AMS interventions in care homes [5, 6]. Moreover, a recent study found six proxy indicators, which had been developed for antimicrobial prescribing in primary care, potentially useful for estimating appropriateness of antimicrobial prescribing in care homes [35]. Additionally, current criteria for appropriateness of antimicrobial prescribing, such as Loeb minimum criteria or Van Buul algorithms, could also be updated and assessed in future research. Regarding measuring AMR, reliable guidelines for antimicrobial susceptibility testing could be followed [36]. Development of antibiograms for care home AMS has also been advocated due to the potential in monitoring resistance and guiding appropriate antimicrobial prescribing [37]. Additionally, several studies have attempted to measure mortality related to infection by referring to the International Classification of Diseases codes, or by establishing criteria for infection-related mortality with the involvement of infectious disease specialists [38, 39]. Nevertheless, any OMIs developed should be assessed for measurement properties before being used in trials.

As the COS represents the minimum that should be used in research [8], trialists may consider exploring other outcomes, such as those included after the outcome Delphi survey but excluded after the online consensus exercise. The aim of the online consensus exercise was to agree on three to seven outcomes in the COS, to minimise the use of multiple outcomes in a trial [40]. However, as the size of the outcome Delphi panel was larger than that of the online consensus panel, it is reasonable to consider the use of any of the excluded outcomes, depending on the focus of a trial. For example, ‘Antimicrobial courses adherent to guidelines’ may be considered when the outcome ‘Appropriateness of antimicrobial prescribing’ in the COS cannot be used.

Although the aim of this study was to develop a COS for use in clinical trials aimed at improving AMS in care homes, this COS may be used to monitor AMS programmes implemented in care homes as part of everyday practice. Current guidelines for implementation of care home AMS support monitoring antimicrobial use by measuring two outcomes, the number of antimicrobial courses and days of therapy, which are also included in the COS [4, 41]. In addition, data describing appropriateness of antimicrobial prescribing and AMR in care homes, which are also listed in the COS, have been recommended to be collected to evaluate the effectiveness of AMS programmes [37, 41]. Outcomes monitoring the safety of AMS programmes have not yet been included in these guidelines; therefore, the outcome ‘Mortality related to infection’ may be used for such purposes. Furthermore, our previous qualitative study of AMS in care homes reported another potential approach to reduce antimicrobial prescribing in care homes: aspects of patient care for prevention and treatment of infection (e.g. hydration to prevent or alleviate urinary tract infection, mouth care to avoid respiratory tract infection) [7]. Such practice was described in a feasibility study of AMS in care homes: the decision-making algorithm guided staff to focus more on non-pharmacological patient care for infection treatment and prevention (e.g. encouraging fluid intake) before consulting general practitioners for treatment [42]. Vaccination and good infection prevention and control practices can reduce the prevalence of infection in care homes, which may subsequently reduce the need for antimicrobial consumption [43]. Indeed, these practices in care homes have been enhanced due to the ongoing COVID-19 pandemic [44].

This study had several strengths. The process of development of this COS followed a well-established methodology developed by the COMET and COSMIN initiatives. The study findings are robust as the two Delphi exercises involved 82 and 59 participants, respectively, from four continents with various professional backgrounds. Moreover, the response rates of all Delphi rounds were high (larger than 85%). By selecting the more stringent criteria for inclusion than those in previous studies [8], outcomes included in the COS represented higher levels of consensus across the Delphi panel. Additionally, the COS comprised five outcomes along with two recommended OMIs, which may be practical for measurement in a trial. However, the five excluded outcomes after the online consensus exercise may still have a place in some trials.

The study also had a number of limitations. The recruitment rates in the two Delphi consensus exercises were low (36% and 56.2%, respectively). Participation of care home managers/staff and representatives of older people in the two Delphi surveys had been actively sought, but few of them participated in the study. Although there was no discrepancy between these participants and other stakeholder groups in rating outcomes and OMIs included in the COS, their opinions were in the minority in these consensus exercises. In addition, it might be expected that the final outcomes included in the COS could have been different if a face-to-face meeting had taken place instead of the online consensus exercise. The size of the online consensus exercise panel was small, and all participants were from NI; therefore, the findings may be limited to this context. Regarding selection of OMIs for the COS, a limited number of OMIs were identified and no OMIs for the two outcomes ‘Rate of antimicrobial resistance’ and ‘Mortality related to infection’ were selected for the consensus procedure. The literature searches also excluded non-English articles; however, other approaches were exploited to search for potential OMIs, including screening of reference lists of included articles and discussion with experts.

## Conclusion

A COS for use in trials aimed at improving AMS in care homes was developed. We recommend the use of this COS along with two selected OMIs in future trials to ensure consistency of measurement. We hope that future trials to improve AMS in care homes will use this COS to measure the effectiveness and safety of interventions. Thereafter, trial findings can be synthesised to produce better evidence in systematic reviews or meta-analyses.

## Supplementary Information

**Additional file 1:** Supplementary data for development of a core outcome set for clinical trials aimed at improving antimicrobial stewardship in care homes; **Table S1:** Inventory of outcomes by categories before the three-round Delphi exercise; **Table S2:** Search strategies to identify studies reporting outcome measurement instruments for antimicrobial stewardship; **Table S3:** Summary of the percentage of Delphi panel members rating each outcome during the outcome Delphi consensus survey; **Table S4:** Distribution of importance based on the scale used for outcomes after the online consensus exercise; **Table S5:** Summary of 55 included studies; **Table S6:** Methodological quality of studies aimed at developing OMIs relevant to antimicrobial prescribing in care homes; **Table S7:** Summary of quality assessment of ‘objective’ outcome measurement instruments; **Table S8:** Summary of quality assessment of ‘subjective’ outcome measurement instruments; **Table S9:** Quality assessment of feasibility aspects of outcome measurement instruments for the Core Outcome Set; **Table S10:** Distribution of agreement levels for each OMI after the OMI Delphi consensus survey.

## Data Availability

The datasets generated and/or analysed during the current study are not publicly available to preserve the anonymity of the participants but are available from the corresponding author on reasonable request.

## Abbreviations

AMR: Antimicrobial resistance
AMS: Antimicrobial stewardship
COMET: Core Outcome Measures in Effectiveness Trials
COS: Core outcome set
COSMIN: Consensus-based standards for the selection of health measurement instruments
NGT: Nominal Group Technique
NI: Northern Ireland
PSG: Project steering group

## References

[1] Lim CJ, Kong DC, Stuart RL (2014). Reducing inappropriate antibiotic prescribing in the residential care setting: current perspectives. Clin Interv Aging.

[2] Peron EP, Hirsch AA, Jury LA, Jump RL, Donskey CJ (2013). Another setting for stewardship: high rate of unnecessary antimicrobial use in a veterans affairs long-term care facility. J Am Geriatr Soc.

[3] van Buul LW, van der Steen JT, Veenhuizen RB, Achterberg WP, Schellevis FG, Essink RTGM (2012). Antibiotic use and resistance in long term care facilities. J Am Med Dir Assoc.

[4] Barlam TF, Cosgrove SE, Abbo LM, Macdougall C, Schuetz AN, Septimus EJ (2016). Executive summary: implementing an antibiotic stewardship program: Guidelines by the Infectious Diseases Society of America and the Society for Healthcare Epidemiology of America. Clin Infect Dis.

[5] Feldstein D, Sloane PD, Feltner C (2018). Antibiotic stewardship programs in nursing homes: a systematic review. J Am Med Dir Assoc.

[6] Nguyen HQ, Tunney MM, Hughes CM (2019). Interventions to improve antimicrobial stewardship for older people in care homes: a systematic review. Drugs Aging.

[7] Nguyen HQ, Bradley DT, Tunney MM, Hughes CM (2020). Antimicrobial stewardship in care homes: outcomes of importance to stakeholders. J Hosp Infect.

[8] Williamson PR, Altman DG, Bagley H, Barnes KL, Blazeby JM, Brookes ST, et al. The COMET Handbook: Version 1.0. Trials. 2017;18:280. 10.1186/s13063-017-1978-4 PMC5499094 28681707

[9] Williamson PR, Altman DG, Blazeby JM, Clarke M, Devane D, Gargon E (2012). Developing core outcome sets for clinical trials: Issues to consider. Trials.

[10] Prinsen CAC, Vohra S, Rose MR, Boers M, Tugwell P, Clarke M (2016). How to select outcome measurement instruments for outcomes included in a “Core Outcome Set”—A practical guideline. Trials.

[11] McMillan SS, King M, Tully MP (2016). How to use the nominal group and Delphi techniques. Int J Clin Pharm.

[12] Belan M, Thilly N, Pulcini C (2020). Antimicrobial stewardship programmes in nursing homes: a systematic review and inventory of tools. J Antimicrob Chemother.

[13] Terwee CB, Prinsen CAC, Chiarotto A, Westerman MJ, Patrick DL, Alonso J (2018). COSMIN methodology for evaluating the content validity of patient-reported outcome measures: a Delphi study. Qual Life Res.

[14] Prinsen CAC, Mokkink LB, Bouter LM, Alonso J, Patrick DL, de Vet HCW (2018). COSMIN guideline for systematic reviews of patient-reported outcome measures. Qual Life Res.

[15] Mokkink LB, de Vet HCW, Prinsen CAC, Patrick DL, Alonso J, Bouter LM (2018). COSMIN risk of bias checklist for systematic reviews of patient-reported outcome measures. Qual Life Res.

[16] Mylotte JM, Neff M (2003). Trends in antibiotic use and cost and influence of case-mix and infection rate on antibiotic-prescribing in a long-term care facility. Am J Infect Control.

[17] Mylotte JM, Keagle J (2005). Benchmarks for antibiotic use and cost in long-term care. J Am Geriatr Soc.

[18] Mylotte JM (1999). Antimicrobial prescribing in long-term care facilities: prospective evaluation of potential antimicrobial use and cost indicators. Am J Infect Control.

[19] Kabbani S, Palms DL, Bartoces M, Marek J, Stone ND, Hicks LA (2019). Potential utility of pharmacy data to measure antibiotic use in nursing homes. Infect Control Hosp Epidemiol.

[20] Fleet E, Rao GG, Patel B, Cookson B, Charlett A, Bowman C (2014). Impact of implementation of a novel antimicrobial stewardship tool on antibiotic use in nursing homes: a prospective cluster randomized control pilot study. J Antimicrob Chemother.

[21] Stuart RL, Orr E, Kotsanas D, Gillespie EE (2015). A nurse-led antimicrobial stewardship intervention in two residential aged care facilities. Healthc Infect.

[22] Mylotte JM (1996). Measuring antibiotic use in a long-term care facility. Am J Infect Control.

[23] Loeb M, Bentley DW, Bradley S, Crossley K, Garibaldi R, Gantz N (2001). Development of minimum criteria for the initiation of antibiotics in residents of long-term–care facilities: results of a consensus conference. Infect Control Hosp Epidemiol.

[24] Stone ND, Ashraf MS, Calder J, Crnich CJ, Crossley K, Drinka PJ (2012). Surveillance definitions of infections in long-term care facilities: revisiting the McGeer criteria. Infect Control Hosp Epidemiol.

[25] McGeer A, Campbell B, Emori TG, Hierholzer WJ, Jackson MM, Nicolle LE (1991). Definitions of infection for surveillance in long-term care facilities. Am J Infect Control.

[26] Crnich CJ, Drinka P (2014). Improving the management of urinary tract infections in nursing homes: It’s time to stop the tail from wagging the dog. Ann Long-Term Care.

[27] van Buul LW, Veenhuizen RB, Achterberg WP, Schellevis FG, Essink RTGM, de Greeff SC (2015). Antibiotic prescribing in Dutch nursing homes: How appropriate is it?. J Am Med Dir Assoc.

[28] Hanlon JT, Schmader KE, Samsa GP, Weinberger M, Uttech KM, Lewis IK (1992). A method for assessing drug therapy appropriateness. J Clin Epidemiol.

[29] Samsa GP, Hanlon JT, Schmader KE, Weinberger M, Clipp EC, Uttech KM (1994). A summated score for the medication appropriateness index: development and assessment of clinimetric properties including content validity. J Clin Epidemiol.

[30] Morris AM, Brener S, Dresser L, Daneman N, Dellit TH, Avdic E (2012). Use of a structured panel process to define quality metrics for antimicrobial stewardship programs. Infect Control Hosp Epidemiol.

[31] Furuno JP, Comer AC, Johnson JK, Rosenberg JH, Moore SL, MacKenzie TD (2014). Using antibiograms to improve antibiotic prescribing in skilled nursing facilities. Infect Control Hosp Epidemiol.

[32] Doernberg SB, Dudas V, Trivedi KK (2015). Implementation of an antimicrobial stewardship program targeting residents with urinary tract infections in three community long-term care facilities: A quasi-experimental study using time-series analysis. Antimicrob Resist Infect Control.

[33] Laxminarayan R, Klugman KP (2011). Communicating trends in resistance using a drug resistance index. BMJ Open.

[34] Loeb M, Brazil K, Lohfeld L, McGeer A, Simor A, Stevenson K (2005). Effect of a multifaceted intervention on number of antimicrobial prescriptions for suspected urinary tract infections in residents of nursing homes: cluster randomised controlled trial. Br Med J.

[35] Simon M, Pereira O, Hulscher ME, Schouten J, Thilly N, Pulcini C. Quantity metrics and proxy indicators to estimate the volume and appropriateness of antibiotics prescribed in French nursing homes: a cross-sectional observational study based on 2018 reimbursement data. Clin Infect Dis. 2020 (in press). 10.1093/cid/ciaa1221 32822471

[36] Clinical Institute Laboratory Standards. Microbiology: Standards. 2020. https://clsi.org/standards/products/microbiology/documents/. Accessed 27 Aug 2020.

[37] Jump RLP, Gaur S, Katz MJ, Crnich CJ, Dumyati G, Ashraf MS (2017). Template for an antibiotic stewardship policy for post-acute and long-term care settings. J Am Med Dir Assoc.

[38] Armstrong GL, Conn LA, Pinner RW (1999). Trends in infectious disease mortality in the United States during the 20th century. JAMA.

[39] Eskesen AN, Belle MA, Blomfeldt A (2018). Predictors of one-year all-cause mortality and infection-related mortality in patients with *Staphylococcus aureus* bacteraemia. Infect Dis (Auckl).

[40] Marcum ZA, Steinman MA (2018). Developing a core outcome set for trials to improve medication use: guidelines or guidance?. J Am Geriatr Soc.

[41] Centers for Disease Control and Prevention. The Core Elements of Antibiotic Stewardship for Nursing Homes. Atlanta; 2015. http://www.cdc.gov/longtermcare/index.htm.

[42] Potter R, Campbell A, Ellard DR, Shaw C, Gardner E, Agus A (2019). Multifaceted intervention to reduce antimicrobial prescribing in care homes: a process evaluation of a UK-based non-randomised feasibility study. BMJ Open.

[43] Montoya A, Mody L (2011). Common infections in nursing homes: a review of current issues and challenges. Aging health.

[44] European Centre for Disease Prevention and Control. Infection prevention and control for COVID-19 in healthcare settings - Fourth update. ECDC: Stockholm; 2020. https://www.ecdc.europa.eu/sites/default/files/documents/Infection-prevention-and-control-in-healthcare-settings-COVID-19_4th_update.pdf.

