# Supplementary material for: Development of a core outcome set for clinical trials aimed at improving antimicrobial stewardship in care homes
Source: Antimicrob Resist Infect Control. 2021 Mar 9;10:52. doi: 10.1186/s13756-021-00925-8 (PMC7941135; doi:10.1186/s13756-021-00925-8)
Supplement: Supplementary file 1 — Additional file 1: Supplementary data for development of a core outcome set for clinical trials aimed at improving antimicrobial stewardship in care homes; Table S1: Inventory of outcomes by categories before the three-round Delphi exercise; Table S2: Search strategies to identify studies reporting outcome measurement instruments for antimicrobial stewardship; Table S3: Summary of the percentage of Delphi panel members rating each outcome during the outcome Delphi consensus survey; Table S4: Distribution of importance based on the scale used for outcomes after the online consensus exercise; Table S5: Summary of 55 included studies; Table S6: Methodological quality of studies aimed at developing OMIs relevant to antimicrobial prescribing in care homes; Table S7: Summary of quality assessment of ‘objective’ outcome measurement instruments; Table S8: Summary of quality assessment of ‘subjective’ outcome measurement instruments; Table S9: Quality assessment of feasibility aspects of outcome measurement instruments for the Core Outcome Set; Table S10: Distribution of agreement levels for each OMI after the OMI Delphi consensus survey. [file 13756_2021_925_MOESM1_ESM.doc]

Table S1 Inventory of outcomes by categories

| **Category** | **Outcome** |
| --- | --- |
| Delivery of care | The total number of antimicrobial courses prescribed |
|  | Antimicrobial courses adherent to guidelines |
|  | Appropriateness of antimicrobial prescribing |
|  | Antimicrobials prescribed through a telephone call |
|  | Antimicrobial use in prophylaxis |
|  | Types of antimicrobials |
|  | Days of therapy |
|  | Antimicrobial consumption based on defined daily doses |
| Infection outcome | Rate of antimicrobial resistance |
|  | Rate of *Clostridioides difficile* infection |
|  | Rate of infection complication |
| Hospitalisation | Hospitalisation due to infection |
| Mortality/survival | All-cause mortality |
|  | Mortality related to infection |

Table S2 Search strategies to identify studies reporting outcome measurement instruments for antimicrobial stewardship

| **Database** | **Search strategy** |
| --- | --- |
| Ovid Medline | 1. (antibiotic? or antimicrobial? or antibacterial? or antiinfective? or antiviral? or antifungal? or anti-microbial? or anti-bacterial? or anti-infective? or anti-viral? or anti-fungal?).ti,ab.  2. (care home? or nursing home? or long-term care or LTCF?).ti,ab.  3. 1 and 2  4. ((antibiotic? or antimicrobial? or antibacterial? or antiinfective? or antiviral? or antifungal? or anti-microbial? or anti-bacterial? or anti-infective? or anti-viral? or anti-fungal?) adj2 (stewardship or prescribing)).ti,ab.  5. 3 or 4  6. (metric* or tool* or criteri* or measur* or instrument* or indicator? or outcome?).ti.  7. 5 and 6  8. limit 7 to dt=19460101-20200229  9. limit 8 to english language |
| Ovid Embase | 1. (antibiotic? or antimicrobial? or antibacterial? or antiinfective? or antiviral? or antifungal? or anti-microbial? or anti-bacterial? or anti-infective? or anti-viral? or anti-fungal?).ti,ab.  2. (care home? or nursing home? or long-term care or LTCF?).ti,ab.  3. 1 and 2  4. ((antibiotic? or antimicrobial? or antibacterial? or antiinfective? or antiviral? or antifungal? or anti-microbial? or anti-bacterial? or anti-infective? or anti-viral? or anti-fungal?) adj2 (stewardship or prescribing)).ti,ab.  5. 3 or 4  6. (metric* or tool* or criteri* or measur* or instrument* or indicator? or outcome?).ti.  7. 5 and 6  8. limit 7 to dd=19740101-20200229  9. limit 7 to rd=19740101-20200229  10. 8 or 9  11. limit 10 to english language |
| COSMIN | The following key words were used:  “antibiotics”, “antimicrobial”, “stewardship”, “prescribing”, “resistance”, “infection”, “nursing home”, “care home”, “long-term care” |
| OpenGrey | “antimicrobial stewardship” OR “antibiotic stewardship”  (antibiotic OR antimicrobial) AND (measure OR metric OR tool OR instrument OR criteria) |
| Grey Literature Report | “antimicrobial stewardship” OR “antibiotic stewardship”  antibiotic AND tool  antibiotic AND measure  antibiotic AND metric  antibiotic AND instrument  antibiotic AND criteria  antimicrobial AND measure  antimicrobial AND metric  antimicrobial AND instrument  antimicrobial AND criteria |
| ProQuest Dissertations | ab(antibiotic OR antimicrobial) AND ab(measure OR metric OR tool OR instrument OR criteria) AND (stewardship OR prescribing) |
| World Health Organization | (antibiotic OR antimicrobial) AND (older OR elderly OR "long-term care") |

Table S3 Summary of the percentage of Delphi panel members rating each outcome during the outcome Delphi consensus survey

| **Round 1** | | | | |
| --- | --- | --- | --- | --- |
| **Outcome**  **(n=14)** | **Rating by 82 participants, n (%)** | | | |
|  | **Unimportant** | **Important but not critical** | **Critical** | **Unable to score** |
| ***Delivery of care*** | | | | |
| The total number of antimicrobial courses prescribed | 5 (6.1) | 8 (9.8) | 69 (84.1) | 0 (0.0) |
| Antimicrobial courses adherent to guidelines | 1 (1.2) | 6 (7.3) | 74 (90.2) | 1 (1.2) |
| Appropriateness of antimicrobial prescribing | 1 (1.2) | 12 (14.6) | 68 (82.9) | 1 (1.2) |
| Antimicrobials prescribed through a telephone call | 9 (11.0) | 41 (50.0) | 32 (39.0) | 0 (0.0) |
| Antimicrobial use in prophylaxis | 7 (8.5) | 20 (24.4) | 54 (65.9) | 1 (1.2) |
| Types of antimicrobials | 3 (3.7) | 16 (19.5) | 63 (76.8) | 0 (0.0) |
| Days of therapy | 2 (2.4) | 22 (26.8) | 58 (70.7) | 0 (0.0) |
| Antimicrobial consumption based on defined daily doses | 9 (11.0) | 39 (47.6) | 34 (41.5) | 0 (0.0) |
| ***Infection*** | | | | |
| Rate of antimicrobial resistance | 1 (1.2) | 17 (20.7) | 63 (76.8) | 1 (1.2) |
| Rate of *Clostridioides difficile* infection | 3 (3.7) | 11 (13.4) | 68 (82.9) | 0 (0.0) |
| Rate of infection complication | 5 (6.1) | 27 (32.9) | 50 (61.0) | 0 (0.0) |
| ***Hospital*** | | | | |
| Hospitalisation due to infection | 2 (2.4) | 22 (26.8) | 58 (70.7) | 0 (0.0) |
| ***Mortality/ survival*** | | | | |
| All-cause mortality | 12 (14.6) | 43 (52.4) | 26 (31.7) | 1 (1.2) |
| Mortality related to infection | 6 (7.3) | 23 (28.0) | 52 (63.4) | 1 (1.2) |
| **Round 2** | | | | |
| **Outcome**  **(n=17)** | **Rating by 77 participants, n (%)** | | | |
|  | **Unimportant** | **Important but not critical** | **Critical** | **Unable to score** |
| ***Delivery of care*** | | | | |
| The total number of antimicrobial courses prescribed^*^ | 1 (1.3) | 6 (7.8) | 70 (90.9) | 0 (0.0) |
| Antimicrobial courses adherent to guidelines^*^ | 0 (0.0) | 5 (6.5) | 72 (93.5) | 0 (0.0) |
| Appropriateness of antimicrobial prescribing^*^ | 1 (1.3) | 9 (11.7) | 67 (87.0) | 0 (0.0) |
| Antimicrobials prescribed through a telephone call | 13 (16.9) | 45 (58.4) | 19 (24.7) | 0 (0.0) |
| Antimicrobial use in prophylaxis | 5 (6.5) | 16 (20.8) | 56 (72.7) | 0 (0.0) |
| Types of antimicrobials^*^ | 1 (1.3) | 13 (16.9) | 63 (81.8) | 0 (0.0) |
| Proportion of broad- and narrow-spectrum antimicrobials | 6 (7.8) | 29 (37.7) | 42 (54.5) | 0 (0.0) |
| Days of therapy per 1000 resident-days | 1 (1.3) | 19 (24.7) | 57 (74.0) | 0 (0.0) |
| Antimicrobial consumption based on defined daily doses | 7 (9.1) | 47 (61.0) | 23 (29.9) | 0 (0.0) |
| ***Infection*** | | | | |
| Rate of antimicrobial resistance^*^ | 0 (0.0) | 8 (10.4) | 69 (89.6) | 0 (0.0) |
| Rate of *Clostridioides difficile* infection^*^ | 0 (0.0) | 6 (7.8) | 71 (92.2) | 0 (0.0) |
| Rate of infection complication | 1 (1.3) | 28 (36.4) | 48 (62.3) | 0 (0.0) |
| Use of laboratory tests | 5 (6.5) | 34 (44.2) | 36 (46.8) | 2 (2.6) |
| ***Hospital*** | | | | |
| Hospitalisation due to infection | 1 (1.3) | 21 (27.3) | 55 (71.4) | 0 (0.0) |
| Emergency department visits | 5 (6.5) | 30 (39.0) | 42 (54.5) | 0 (0.0) |
| ***Mortality/ survival*** | | | | |
| All-cause mortality | 12 (15.6) | 53 (68.8) | 11 (14.3) | 1 (1.3) |
| Mortality related to infection | 3 (3.9) | 20 (26.0) | 54 (71.1) | 0 (0.0) |
| **Round 3** | | | | |
| **Outcome**  **(n=11)** | **Rating by 75 participants, n (%)** | | | |
|  | **Unimportant** | **Important but not critical** | **Critical** | **Unable to score** |
| ***Delivery of care*** | | | | |
| Antimicrobials prescribed through a telephone call | 12 (16.0) | 39 (52.0) | 23 (30.7) | 1 (1.3) |
| Antimicrobial use in prophylaxis^*^ | 1 (1.3) | 8 (10.7) | 66 (88.0) | 0 (0.0) |
| Proportion of broad- and narrow-spectrum antimicrobials | 1 (1.3) | 16 (21.3) | 58 (77.3) | 0 (0.0) |
| Days of therapy per 1000 resident-days^*^ | 1 (1.3) | 9 (12.0) | 65 (86.7) | 0 (0.0) |
| Antimicrobial consumption based on defined daily doses | 4 (5.3) | 47 (62.7) | 22 (32.0) | 0 (0.0) |
| ***Infection*** | | | | |
| Rate of infection complication | 1 (1.3) | 21 (28.0) | 53 (70.7) | 0 (0.0) |
| Use of laboratory tests | 4 (5.3) | 33 (44.0) | 38 (50.7) | 0 (0.0) |
| ***Hospital*** | | | | |
| Hospitalisation due to infection^*^ | 0 (0.0) | 13 (17.3) | 62 (82.7) | 0 (0.0) |
| Emergency department visits | 4 (5.3) | 27 (36.0) | 44 (58.7) | 0 (0.0) |
| ***Mortality/ survival*** | | | | |
| All-cause mortality | 11 (14.7) | 52 (69.3) | 11 (14.7) | 1 (1.3) |
| Mortality related to infection^*^ | 2 (2.7) | 9 (12.0) | 63 (84.0) | 1 (1.3) |

^*^Outcomes included after the Delphi survey.

Table S4 Distribution of importance based on the scale used for outcomes after the online consensus exercise

| **Outcome**  **(n=11)** | **Rating by 11 participants, n (%)*** | | |
| --- | --- | --- | --- |
|  | **Unimportant** | **Important but not critical** | **Critical** |
| The total number of antimicrobial courses prescribed | 0 (0.0) | 0 (0.0) | 11 (100.0) |
| Appropriateness of antimicrobial prescribing | 0 (0.0) | 0 (0.0) | 11 (100.0) |
| Days of therapy per 1000 resident-days | 0 (0.0) | 3 (27.3) | 8 (72.7) |
| Mortality related to infection | 0 (0.0) | 3 (27.3) | 8 (72.7) |
| Rate of antimicrobial resistance | 1 (9.1) | 2 (18.2) | 8 (72.7) |
| Hospitalisation due to infection | 1 (9.1) | 3 (27.3) | 7 (63.6) |
| Antimicrobial courses adherent to guidelines | 1 (9.1) | 5 (45.5) | 5 (45.5) |
| Rate of *Clostridioides difficile* infection | 1 (9.1) | 5 (45.5) | 5 (45.5) |
| Antimicrobial use in prophylaxis | 0 (0.0) | 7 (63.6) | 4 (36.4) |
| Types of antimicrobials | 2 (18.2) | 5 (45.5) | 4 (36.4) |

^*^ Some percentages do not add to 100% due to rounding.

Table S5 Summary of 55 included studies

| **Article**  **(Author, year)** | **Study design** | **OMI mentioned relevant to the Core Outcome Set** | **Study aim** |
| --- | --- | --- | --- |
| Mylotte 1996* | OMI development | Number of residents treated with antibiotics | To compare various measures of antibiotic use and to correlate these measures with febrile morbidity and Foley catheter use |
| Mylotte 1999* | OMI development | Incidence of antibiotic use; days of therapy | To develop measures for monitoring antimicrobial use and cost in 4 care homes |
| Mylotte 2003* | OMI development | Incidence of antibiotic use; days of therapy | To assess the utility of the antibiotic use and cost indicators and to determine the correlation of case-mix and these indicators |
| Mylotte 2005* | OMI development | Incidence of antibiotic use; days of therapy | To determine whether the antibiotic use and cost indicators may be useful as benchmarks and to evaluate the association between functional status, infection occurrence, and antibiotic use |
| Kabbani 2019* | OMI development | Number of antibiotic transactions | To assess the feasibility of pharmacy transaction data to measure antibiotic days-of-therapy and antibiotic starts in nursing homes |
| Loeb 2001* | OMI development | Minimum criteria for initiating antibiotic therapy (Loeb criteria) | To establish the minimum criteria for the initiation of antibiotics for UTIs, RTIs, SSTIs and fever amongst care homes residents |
| McGeer 1991* | OMI development | Criteria for diagnosing infection (McGeer criteria) | To define infection for surveillance in care homes |
| Stone 2012* | OMI development | Criteria for diagnosing infection (revised McGeer criteria) | To update the McGeer criteria by an evidence-based structured review of the literature in addition to consensus opinions |
| Hanlon 1992* | OMI development | Medication appropriateness index | To evaluate the inter-rater reliability of the medication appropriateness index |
| Samsa 1994* | OMI development | Medication appropriateness index | To develop and validate a weighting scheme for a single summated Medication appropriateness index score per medication |
| Fleet 2014* | RCT | Point prevalence of antibiotic prescribing | To evaluate the effectiveness of an AMS intervention in nursing homes, aimed at nursing staff |
| Loeb 2005* | RCT | Revised Loeb criteria for UTIs; antibiotic courses per 1000 resident-days; overall mortality | To evaluate the effectiveness of an AMS intervention focusing on suspected UTIs in nursing homes |
| Monette 2007 | RCT | Antibiotic non-compliant to guidelines | To evaluate the effectiveness of an AMS intervention in nursing homes, aimed at physicians |
| Naughton 2001 | RCT | Antibiotics compliant to guidelines | To evaluate the effectiveness of two AMS interventions focusing on nursing home-acquired pneumonia in nursing homes |
| Pettersson 2011 | RCT | Number of specific antibiotics for UTIs in women | To evaluate the effectiveness of an AMS intervention focusing on UTIs in nursing homes |
| Pasay 2019 | RCT | Number of UTI systemic antimicrobial prescriptions per 1000 resident-days | To evaluate the effectiveness of an AMS intervention focusing on UTIs in nursing homes |
| Van Buul 2015* | Non-RCT | Number of antimicrobial prescriptions per 1000 resident-days; Van Buul algorithms | To evaluate the effectiveness of an AMS intervention in nursing homes |
| Cooper 2017 | Non-RCT | Loeb criteria; McGeer criteria; revised McGeer criteria | To evaluate the effectiveness of an AMS intervention focusing on suspected UTIs in care homes |
| Doernberg 2015* | Non-RCT | Antibiotic starts per 1000 resident-days; Loeb criteria; number of specific resistant organisms | To evaluate the effectiveness of an AMS intervention focusing on UTIs in care homes |
| Furuno 2014* | Non-RCT | Number of specific susceptible organisms | To evaluate the effectiveness of an AMS intervention in nursing homes using antibiograms |
| Gugkaeva 2012 | Non-RCT | Antibiotics compliant to guidelines | To evaluate the effectiveness of an AMS intervention in care homes, aimed at pharmacists |
| Jump 2012 | Non-RCT | Days of therapy per 1000 resident-days | To evaluate the effectiveness of an AMS intervention in care homes |
| Linnebur 2011 | Non-RCT | Mean of days on antibiotics for pneumonia; overall mortality rate in percentage | To evaluate the effectiveness of an AMS intervention focusing on nursing home-acquired pneumonia in nursing homes |
| McMaughan 2016 | Non-RCT | Percentage of antibiotic prescriptions written for suspected asymptomatic bacteriuria | To evaluate the effectiveness of an AMS intervention focusing on suspected UTIs in nursing homes |
| Sloane 2014 | Non-RCT | Number of systemic antibiotic prescriptions per 1000 resident-days | To evaluate the effectiveness of an AMS intervention in care homes |
| Stuart 2015* | Non-RCT | Total counts of antibiotic courses; days of therapy per 1000 occupied bed days | To evaluate the effectiveness of an AMS intervention in residential homes, aimed at nurses |
| Zabarsky 2008 | Non-RCT | Antimicrobial days of therapy per 1000 patient-days | To evaluate the effectiveness of an AMS intervention focusing on asymptomatic bacteriuria in care homes |
| Zimmerman 2014 | Non-RCT | Number of antibiotic prescriptions per 1,000 resident-days | To evaluate the effectiveness of an AMS intervention in nursing homes |
| Hutt 2006 | Non-RCT | Antibiotics compliant to guidelines | To evaluate the effectiveness of an AMS intervention focusing on nursing home-acquired pneumonia in nursing homes |
| Kassett 2016 | Non-RCT | Ciprofloxacin rate and proportion; days of therapy | To evaluate the effectiveness of an AMS intervention in care homes |
| Rummukainen 2012 | Non-RCT | Number of patients treated with antibiotics | To evaluate the effectiveness of an AMS intervention focusing on UTIs in care homes |
| Schwartz 2007 | Non-RCT | Antimicrobial starts per 1000 patient days; antimicrobial days per 1000 patient days | To evaluate the effectiveness of an AMS intervention in care homes, aimed at physicians |
| Trautner 2015 | Non-RCT | Number of cases of overtreatment (or undertreatment) per 1000 bed days | To evaluate the effectiveness of an AMS intervention focusing on urinary catheter associated asymptomatic bacteriuria in care homes |
| Daneman 2015 | Prospective | Antibiotic use-days per 1000 resident-days | To examine the association between high antibiotic use antibiotic-related adverse outcomes for individual residents |
| Juthani-Mehta 2007* | Prospective | McGeer criteria, Loeb criteria, revised Loeb criteria | To evaluate the reliability of criteria for UTI with laboratory evidence of UTI |
| Van Buul 2015* | Prospective | Van Buul algorithm to evaluate appropriateness of initiating or withholding antibiotics | To investigate the appropriateness of initiating or withholding antibiotics for nursing home residents with RTI, SSTI and UTI |
| Olsho 2013* | Cross-sectional | Loeb criteria | To examine the relationship between nursing home prescriber adherence to the Loeb criteria and antibiotic prescribing rates |
| Eure 2017* | Cross-sectional | McGeer criteria, Loeb criteria, Crnich algorithm | To assess the appropriateness of initiating antibiotics in nursing home residents with UTI using 3 published algorithms |
| Daneman 2013 | Retrospective analysis | Antibiotic treatment duration | To describe the variability in the duration of antibiotic treatment courses in care homes and to determine whether this variability is influenced by prescriber preference |
| Fridkin 2019 | Retrospective analysis | Number of specific susceptible organisms | To produce a useful annual antibiogram that nursing homes can use in their antimicrobial stewardship programs |
| Penna 2020 | Retrospective analysis | Revised McGeer criteria | To evaluate the impact of using a more sensitive, modified acute change in mental status criterion in infection surveillance definitions |
| Crnich 2014* | OMI suggestion | Crnich algorithm for evaluation and treatment of suspected UTIs in nursing homes | To suggest an algorithm to diagnose a UTI and start antibiotic therapy based on published guidelines and experts’ opinions |
| Laxminarayan 2011* | OMI suggestion | Drug resistance index | To develop a method for aggregating bacterial resistance to multiple antibiotics in hospital settings |
| Morris 2012* | Consensus | Days of therapy; number of patients with specific resistant organisms; mortality related to antimicrobial-resistant organisms | To use a structured panel process to define quality improvement metrics for evaluating AMS programs in hospital settings that also have the potential to be used as part of public reporting efforts |
| Taylor 2001* | OMI assessment | Medication appropriateness index | To evaluate the reliability of the modified Medication appropriateness index instrument for inpatients to assess antimicrobial therapy |
| Kassam 2003* | OMI assessment | Medication appropriateness index | To evaluate the reliability of the modified Medication appropriateness index instrument in the community pharmacy setting |
| Nace 2014* | Perspective | McGeer criteria, revised McGeer criteria, Loeb criteria | To discuss clinical uncertainties in the approach to care home residents with suspected UTI |
| Crnich 2015 | Review | Crnich algorithm; antibiograms and resistance patterns | To summarise the findings of AMS in nursing homes and to present ways in which AMS can be implemented and optimized |
| Genao 2012 | Review | McGeer criteria, revised Loeb criteria | To outline the characteristics, diagnosis, laboratory assessment, and management of symptomatic UTI for care home residents |
| Mylotte 2016 | Review | Incidence of antibiotic use; days of therapy | To provide a review of studies that have specifically focused on development of metrics for antibiotic use in care homes |
| Rhee 2014 | Review | McGeer criteria, revised McGeer criteria, Loeb criteria | To discuss challenges and possibility of implementing AMS in care homes |
| CDC 2015 | Guidelines | antibiotic starts per 1000 resident-days; point prevalence of antibiotic use; days of therapy | To provide core elements to implement AMS for nursing homes |
| Jump 2017 | Guidelines | Antibiotic starts per 1000 resident-days; days of therapy; number of resistant isolates | To help care homes implement an AMS policy that will meet or exceed the Centers for Medicare & Medicaid services requirements |
| Washington & Idaho State Departments of Health ,year? | Guidelines | Number of resistant isolates; point prevalence of antibiotic use; antibiotic starts per 1000 resident-days; days of therapy | To provide nursing homes with guidance and tools to create a framework and strategic plan for implementing an AMS program tailored to their own unique characteristics |
| WHO 2018 | Guidelines | Days of therapy; antibiotic-resistant organisms | To develop evidence-informed options for European countries to consider AMS in care homes |

* Studies included in the OMI quality assessment.

AMS, antimicrobial stewardship; CDC, Centers for Disease Control and Prevention; OMI, outcome measurement instrument; RCT, randomised controlled trial; RTI, respiratory tract infection; SSTI, Skin and soft tissue infection; UTI, urinary tract infection; WHO, World Health Organisation.

**References:**

1.Mylotte JM. Measuring antibiotic use in a long-term care facility. Am J Infect Control 1996; 24:174–179.

2. Mylotte JM. Antimicrobial prescribing in long-term care facilities: Prospective evaluation of potential antimicrobial use and cost indicators. Am J Infect Control 1999; 27:10–19.

3. Mylotte JM, Neff M. Trends in antibiotic use and cost and influence of case-mix and infection rate on antibiotic-prescribing in a long-term care facility. Am J Infect Control 2003; 31:18–25.

4. Mylotte JM, Keagle J. Benchmarks for antibiotic use and cost in long-term care. J Am Geriatr Soc 2005; 53:1117–1122.

5. Kabbani S, Palms DL, Bartoces M, et al. Potential utility of pharmacy data to measure antibiotic use in nursing homes. Infect Control Hosp Epidemiol 2019; 40:819–820.

6. Loeb M, Bentley DW, Bradley S, et al. Development of Minimum Criteria for the Initiation of Antibiotics in Residents of Long-Term–Care Facilities: Results of a Consensus Conference. Infect Control Hosp Epidemiol 2001; 22:120–124.

7. McGeer A, Campbell B, Emori TG, et al. Definitions of infection for surveillance in long-term care facilities. Am J Infect Control 1991; 19:1–7.

8. Stone ND, Ashraf MS, Calder J, et al. Surveillance Definitions of Infections in Long-Term Care Facilities: Revisiting the McGeer Criteria. Infect Control Hosp Epidemiol 2012; 33:965–977.

9. Hanlon JT, Schmader KE, Samsa GP, et al. A method for assessing drug therapy appropriateness. J Clin Epidemiol 1992; 45:1045–1051.

10. Samsa GP, Hanlon JT, Schmader KE, et al. A summated score for the medication appropriateness index: development and assessment of clinimetric properties including content validity. J Clin Epidemiol 1994; 47:891–896.

11. Fleet E, Rao GG, Patel B, et al. Impact of implementation of a novel antimicrobial stewardship tool on antibiotic use in nursing homes: A prospective cluster randomized control pilot study. J Antimicrob Chemother 2014; 69:2265–2273.

12. Loeb M, Brazil K, Lohfeld L, et al. Effect of a multifaceted intervention on number of antimicrobial prescriptions for suspected urinary tract infections in residents of nursing homes: Cluster randomised controlled trial. Br Med J 2005; 331:669–672.

13. Monette J, Miller MA, Monette M, et al. Effect of an educational intervention on optimizing antibiotic prescribing in long-term care facilities. J Am Geriatr Soc 2007; 55:1231–1235.

14. Naughton B, Mylotte J, Ramadan F, Karuza J, Priore R. Antibiotic Use, Hospital Admissions, and Mortality Before and After Implementing Guidelines for Nursing Home–Acquired Pneumonia. J Am Geriatr Soc 2001; 49:1020–1024.

15. Pettersson E, Vernby Å, Mölstad S, Lundborg CS. Can a multifaceted educational intervention targeting both nurses and physicians change the prescribing of antibiotics to nursing home residents? A cluster randomized controlled trial. J Antimicrob Chemother 2011; 66:2659–2666.

16. Pasay DK, Guirguis MS, Shkrobot RC, et al. Antimicrobial stewardship in rural nursing homes: Impact of interprofessional education and clinical decision tool implementation on urinary tract infection treatment in a cluster randomized trial. Infect Control Hosp Epidemiol 2019; 40:432–437.

17. van Buul LW, van der Steen JT, Achterberg WP, et al. Effect of tailored antibiotic stewardship programmes on the appropriateness of antibiotic prescribing in nursing homes. J Antimicrob Chemother 2015; 70:2153–2162.

18. Cooper DL, Titler M, Struble L, Redman R. A multifaceted, evidence-based program to reduce inappropriate antibiotic treatment of suspected urinary tract infections. Ann Long-Term Care 2017; 25:36–43.

19. Doernberg SB, Dudas V, Trivedi KK. Implementation of an antimicrobial stewardship program targeting residents with urinary tract infections in three community long-term care facilities: A quasi-experimental study using time-series analysis. Antimicrob Resist Infect Control 2015; 4:1–8.

20. Furuno JP, Comer AC, Johnson JK, et al. Using Antibiograms to Improve Antibiotic Prescribing in Skilled Nursing Facilities. Infect Control Hosp Epidemiol 2014; 35:S56–S61.

21. Gugkaeva Z, Franson M. Pharmacist-led model of antibiotic stewardship in a long-term care facility. Ann Long-Term Care 2012; 20:2–6.

22. Jump RLP, Olds DM, Seifi N, et al. Effective antimicrobial stewardship in a long-term care facility through an infectious disease consultation service: keeping a LID on antibiotic use. Infect Control Hosp Epidemiol 2012; 33:1185–1192.

23. Linnebur SA, Fish DN, Ruscin JM, et al. Impact of a multidisciplinary intervention on antibiotic use for nursing home-Acquired Pneumonia. Am J Geriatr Pharmacother 2011; 9:442-450.e1.

24. McMaughan DK, Nwaiwu O, Zhao H, et al. Impact of a decision-making aid for suspected urinary tract infections on antibiotic overuse in nursing homes. BMC Geriatr 2016; 16:1–9.

25. Sloane PD, Zimmerman S, Reed D, et al. Antibiotic Prescribing in 4 Assisted-Living Communities: Incidence and Potential for Improvement. Infect Control Hosp Epidemiol 2014; 35:S62–S68.

26. Stuart RL, Orr E, Kotsanas D, Gillespie EE. A nurse-led antimicrobial stewardship intervention in two residential aged care facilities. Healthc Infect 2015; 20:4–6.

27. Zabarsky TF, Sethi AK, Donskey CJ. Sustained reduction in inappropriate treatment of asymptomatic bacteriuria in a long-term care facility through an educational intervention. Am J Infect Control 2008; 36:476–480.

28. Zimmerman S, Sloane PD, Bertrand R, et al. Successfully reducing antibiotic prescribing in nursing homes. J Am Geriatr Soc 2014; 62:907–912.

29. Hutt E, Ruscin JM, Corbett K, et al. A multifaceted intervention to implement guidelines improved treatment of nursing home-acquired pneumonia in a State Veterans Home. J Am Geriatr Soc 2006; 54:1694–1700.

30. Kassett N, Sham R, Aleong R, Yang D, Kirzner M, Craft A. Impact of antimicrobial stewardship on physician practice in a geriatric facility. Can J Hosp Pharm 2016; 69:460–465.

31. Rummukainen ML, Jakobsson A, Matsinen M, et al. Reduction in inappropriate prevention of urinary tract infections in long-term care facilities. Am J Infect Control 2012; 40:711–714.

32. Schwartz DN, Abiad H, DeMarais PL, et al. An educational intervention to improve antimicrobial use in a hospital-based long-term care facility. J Am Geriatr Soc 2007; 55:1236–1242.

33. Trautner BW, Grigoryan L, Petersen NJ, et al. Effectiveness of an antimicrobial stewardship approach for urinary catheter-associated asymptomatic bacteriuria. JAMA Intern Med 2015; 175:1120–1127.

34. Daneman N, Bronskill SE, Gruneir A, et al. Variability in antibiotic use across nursing homes and the risk of antibiotic-related adverse outcomes for individual residents. JAMA Intern Med 2015; 175:1331–1339.

35. Juthani-Mehta M, Tinetti M, Perrelli E, Towle V, Van Ness PH, Quagliarello V. Diagnostic accuracy of criteria for urinary tract infection in a cohort of nursing home residents. J Am Geriatr Soc 2007; 55:1072–1077.

36. van Buul LW, Veenhuizen RB, Achterberg WP, et al. Antibiotic prescribing in Dutch nursing homes: How appropriate is it? J Am Med Dir Assoc 2015; 16:229–237.

37. Olsho LEW, Bertrand RM, Edwards AS, et al. Does Adherence to the Loeb Minimum Criteria Reduce Antibiotic Prescribing Rates in Nursing Homes? J Am Med Dir Assoc 2013; 14:309.e1-309.e7.

38. Eure T, Laplace LL, Melchreit R, et al. Measuring Antibiotic Appropriateness for Urinary Tract Infections in Nursing Home Residents. Infect Control Hosp Epidemiol 2017; 38:998–1001.

39. Brown KA, Khanafer N, Daneman N, Fisman DN. Meta-Analysis of Antibiotics and the Risk of Community-Associated Clostridium difficile Infection. Antimicrob Agents Chemother 2013; 57:2326–2332.

40. Fridkin SK, Pack J, Licitra G, et al. Creating reasonable antibiograms for antibiotic stewardship programs in nursing homes: Analysis of 260 facilities in a large geographic region, 2016-2017. Infect Control Hosp Epidemiol 2019; 40:839–846.

41. Penna AR, Sancken CL, Stone ND, et al. Documentation of acute change in mental status in nursing homes highlights opportunity to augment infection surveillance criteria. Infect Control Hosp Epidemiol 2020; 41:848–850.

42. Crnich CJ, Drinka P. Improving the management of urinary tract infections in nursing homes: It’s time to stop the tail from wagging the dog. Ann Long-Term Care 2014; 22:32–36.

43. Laxminarayan R, Klugman KP. Communicating trends in resistance using a drug resistance index. BMJ Open 2011; 1:e000135.

44. Morris AM, Brener S, Dresser L, et al. Use of a Structured Panel Process to Define Quality Metrics for Antimicrobial Stewardship Programs. Infect Control Hosp Epidemiol 2012; 33:500–506.

45. Taylor CT, Stewart LM, Byrd DC, Church CO. Reliability of an instrument for evaluating antimicrobial appropriateness in hospitalized patients. Am J Heal Pharm 2001; 58:242–246.

46. Kassam R, Martin LG, Farris KB. Reliability of a modified medication appropriateness index in community pharmacies. Ann Pharmacother 2003; 37:40–46.

47. Nace DA, Drinka PJ, Crnich CJ. Clinical Uncertainties in the Approach to Long Term Care Residents With Possible Urinary Tract Infection. J Am Med Dir Assoc 2014; 15:133–139.

48. Crnich CJ, Jump R, Trautner B, Sloane PD, Mody L. Optimizing Antibiotic Stewardship in Nursing Homes: A Narrative Review and Recommendations for Improvement. Drugs and Aging 2015; 32:699–716.

49. Genao L, Buhr GT. Urinary tract infections in older adults residing in long-term care facilities. Ann Long-Term Care 2012; 20:33–38.

50. Mylotte JM. Antimicrobial Stewardship in Long-Term Care: Metrics and Risk Adjustment. J Am Med Dir Assoc 2016; 17:672.e13-672.e18.

51. Rhee SM, Stone ND. Antimicrobial stewardship in long-term care facilities. Infect Dis Clin North Am 2014; 28:237–246.

52. Centers for Disease Control and Prevention. The Core Elements of Antibiotic Stewardship for Nursing Homes. Atlanta: 2015.

53. Jump RLP, Gaur S, Katz MJ, et al. Template for an Antibiotic Stewardship Policy for Post-Acute and Long-Term Care Settings. J Am Med Dir Assoc 2017; 18:913–920.

54. Washington & Idaho State Departments of Health. JumpStart Stewardship: Implementing Antibiotic Stewardship in Nursing Homes.

55. Evidence brief for policy. Antibiotic prescribing in long-term care facilities for the elderly. Copenhagen: 2018.

Table S6 Methodological quality of studies aimed at developing OMIs relevant to antimicrobial prescribing in care homes

| **Study** | **OMI developed** | **Section assessed** | **Overall quality** |
| --- | --- | --- | --- |
| Kabbani 2019 | Number of antimicrobial transactions | OMI development, hypotheses testing | Inadequate |
| Mylotte 1996 | Number of residents treated with antimicrobials | OMI development, hypotheses testing | Inadequate |
| Mylotte 1999 | Incidence of antimicrobial use; days of therapy | OMI development, hypotheses testing, responsiveness | Adequate |
| Mylotte 2003 | Incidence of antimicrobial use; days of therapy | Hypotheses testing, responsiveness | Adequate |
| Mylotte 2005 | Incidence of antimicrobial use; days of therapy | Hypotheses testing, responsiveness | Adequate |
| Loeb 2001 | Minimum criteria for initiating antibiotic therapy | OMI development, content validity | Adequate |
| McGeer 1991 | Criteria for diagnosing infection | OMI development, content validity | Adequate |
| Stone 2012 | Criteria for diagnosing infection | OMI development, content validity | Adequate |
| Hanlon 1992 | Medication appropriateness index | OMI development, content validity, reliability | Doubtful |
| Samsa 1994 | Medication appropriateness index | Content validity, reliability | Doubtful |

OMI, outcome measurement instrument

Table S7 Summary of quality assessment of ‘objective’ outcome measurement instruments

| **Outcome measurement instrument (Authors, year)** | **Content validity** | **Feasibility aspects** | **Overall quality of evidence** |
| --- | --- | --- | --- |
| *Outcome ‘The total number of antimicrobial courses prescribed’* | | | |
| Number of antimicrobial courses started per 1000 resident-days | + | + | + |
| Number of antimicrobial transactions per 1000 resident-days | ? | ? | ? |
| Point prevalence of antimicrobial use | ? | + | ? |
| Total number of antimicrobial courses | ? | ? | ? |
| Mean number of residents treated with antimicrobial per month | ? | ? | ? |
| *Outcome ‘Days of therapy per 1000 resident-days’* | | | |
| Rate of antimicrobial days of therapy per 1000 resident-days | + | + | + |
| Antibiotic utilization ratio | ? | + | ? |

+, positive rating; ?, indeterminate rating; –, negative rating

Table S8 Summary of quality assessment of ‘subjective’ outcome measurement instruments

| **Outcome measurement instrument (Authors, year)** | **Content validity** | **Structural validity** | **Internal consistency** | **Reliability** | **Measurement error** | **Hypotheses testing** | **Cross‐cultural validity** | **Criterion validity** | **Responsiveness** | **Feasibility aspects** | **Overall quality of evidence** |
| --- | --- | --- | --- | --- | --- | --- | --- | --- | --- | --- | --- |
| *Outcome ‘Appropriateness of antimicrobial prescribing’* | | | | | | | | | | | |
| Loeb minimum criteria for initiating antibiotic therapy in SSTIs, RTIs, UTIs, fever with unknown infection. | + | NA | NA | - | - | NA | NA | - | - | ? | ? |
| Revisited McGeer criteria for diagnosing infection in SSTIs, RTIs, UTIs, Gastrointestinal Tract Infections and Systemic Infections. | ? | NA | NA | - | - | NA | NA | - | - | ? | - |
| Crnich algorithm for the initiation of antibiotics for UTIs | ? | NA | NA | - | - | NA | NA | - | - | ? | - |
| Van Buul algorithms to evaluate appropriateness of initiating or withholding antibiotics in SSTIs, RTIs, UTIs. | + | NA | NA | - | - | NA | NA | - | - | ? | ? |
| The Medication Appropriateness Index | ? | - | - | + | - | - | NA | - | - | ? | - |
| *Outcome ‘Rate of antimicrobial resistance’* | | | | | | | | | | | |
| Number of cases with specific (non-) resistant organisms | - | NA | NA | - | - | NA | NA | - | - | ? | - |
| Number of specific (non-) resistant isolates/ organisms | - | NA | NA | - | - | NA | NA | - | - | ? | - |
| Drug Resistance Index | - | NA | NA | - | - | NA | NA | - | - | - | - |
| *Outcome ‘Mortality related to infection’* | | | | | | | | | | | |
| Rate of mortality related to infection per 1000 resident-days | - | NA | NA | - | - | NA | NA | - | - | ? | - |
| Proportion of mortality related to infection | - | NA | NA | - | - | NA | NA | - | - | ? | - |

NA, not applicable; RTIs, respiratory tract infections; SSTIs, skin and soft tissue infections; UTIs, urinary tract infections.

+, positive rating; ?, indeterminate rating; –, negative rating

Table S9 Quality assessment of feasibility aspects of outcome measurement instruments for the Core Outcome Set

| **Outcome measurement instrument** | **Clinician’ s comprehensibility** | **Interpretability** | **Ease of administration** | **Length of the instrument** | **Completion time** | **Ease of standardization** | **Type of instrument** | **Required equipment** | **Type of administration** | **Availability in different settings** | **Copyright** | **Requirement for approval** |
| --- | --- | --- | --- | --- | --- | --- | --- | --- | --- | --- | --- | --- |
| *Outcome ‘The total number of antimicrobial courses prescribed’* | | | | | | | | | | | | |
| Number of antimicrobial courses started per 1000 resident-days | Yes | Clear | Straight-forward | NA | Quick | Easy | Records | Not needed | Data count | Yes | NA | NA |
| Number of antimicrobial transactions per 1000 resident-days | Maybe | Not clear | Not clear | NA | Quick | Not clear | Records | Not needed | Data count | Pharmacy data | NA | NA |
| Point prevalence of antimicrobial use | Yes | Easy | Easy | NA | Quick | Easy | Records | Not needed | Data count | Yes | NA | NA |
| Total number of antimicrobial courses | Yes | Easy | Easy | NA | Quick | Not clear | Records | Not needed | Data count | Yes | NA | NA |
| Mean number of residents treated with antimicrobial per month | Not clear | Not clear | Not clear | NA | Quick | Not clear | Records | Not needed | Data count | Yes | NA | NA |
| *Outcome ‘Days of therapy per 1000 patient-days’* | | | | | | | | | | | | |
| Rate of antimicrobial days of therapy per 1000 resident-days | Yes | Easy | Easy | NA | Quick | Easy | Records | Not needed | Not needed | Yes | NA | NA |
| Antibiotic utilization ratio | Not clear | Not clear | Easy | NA | Quick | Easy | Records | Not needed | Not needed | Yes | NA | NA |
| *Outcome ‘Appropriateness of antimicrobial prescribing’* | | | | | | | | | | | | |
| Loeb minimum criteria | Yes | Maybe | Medium | Long | Unclear | Maybe | Minimum criteria | Clinical data | Minimum criteria | Probably | Unclear | Unclear |
| McGeer criteria | Yes | Maybe | Medium | Long | Unclear | Maybe | Definitions | Clinical data | Definitions | Probably | Unclear | Unclear |
| Crnich and Drinka algorithm | Yes | Maybe | Medium | Long | Unclear | Maybe | Algorithm | Clinical data | Algorithm | Probably | Unclear | Unclear |
| Van Buul algorithms | Yes | Maybe | Medium | Long | Unclear | Maybe | Algorithm | Clinical data | Algorithm | Probably | Unclear | Unclear |
| The Medication Appropriateness Index | Yes | Yes | Medium | 10 ques-tions | Medi-um | Maybe | Question-naire | Docu-ments | Question-naire | No | Unclear | Unclear |
| *Outcome ‘Rate of antimicrobial resistance’* | | | | | | | | | | | | |
| Number of cases with specific (non-)resistant organisms | Yes | Yes | Easy | NA | Testingtime: 48-72 hours | Yes | Testing | Lab equip-ment | Sample collection & process-ing | Maybe | NA | NA |
| Number of specific (non-)resistant isolates/ organisms | Yes | Yes | Easy | NA | Testingtime: 48-72 hours | Yes | Testing | Lab equip-ment | Sample collection & process-ing | Maybe | NA | NA |
| Drug Resistance Index | Not clear | Not clear | Medium | NA | Testingtime: 48-72 hours | Yes | Biomarker of potential resistance | Lab equip-ment | Sample collection & process-ing | Maybe | NA | NA |
| *Outcome ‘Mortality related to infection’* | | | | | | | | | | | | |
| Rate of mortality related to infection per 1000 resident-days | Yes | Yes | Not clear | NA | Quick | Easy | Records & judgment required | Death certifi-cate & docu-ments | Data count | Yes | NA | NA |
| Proportion of mortality related to infection | Yes | Yes | Not clear | NA | Quick | Easy | Records & judgment required | Death certifi-cate & docu-ments | Data count | Yes | NA | NA |

NA, not applicable

Table S10 Distribution of agreement levels for each OMI after the OMI Delphi consensus survey

| **Round 1** | | | |
| --- | --- | --- | --- |
| **Outcome and measurement instrument (n=3)** | **Rating by 59 participants, n (%)** | | |
|  | **Agree** | **Disagree** | **Unsure** |
| ***Outcome:*** The total number of antimicrobial courses prescribed  ***OMI:*** Incidence of antimicrobial use | 44 (74.6) | 5 (8.5) | 10 (16.9) |
| ***Outcome:*** Days of therapy per 1000 resident-days  ***OMI:*** Rate of antimicrobial days of therapy per 1000 resident-days | 48 (81.4) | 3 (5.1) | 8 (13.6) |
| ***Outcome:*** Appropriateness of antimicrobial prescribing  ***OMI:*** Appropriateness of initiating antimicrobials by Buul *et al*. | 38 (64.4) | 6 (10.2) | 15 (25.4) |
| **Round 2** | | | |
| **Outcome and measurement instrument (n=3)** | **Rating by 54 participants, n (%)** | | |
|  | **Agree** | **Disagree** | **Unsure** |
| ***Outcome:*** The total number of antimicrobial courses prescribed  ***OMI:*** Incidence of antimicrobial use | 47 (87.0) | 4 (7.4) | 3 (5.6) |
| ***Outcome:*** Days of therapy per 1000 resident-days  ***OMI:*** Rate of antimicrobial days of therapy per 1000 resident-days | 51 (94.4) | 1 (1.9) | 2 (3.7) |
| ***Outcome:*** Appropriateness of antimicrobial prescribing  ***OMI:*** Appropriateness of initiating antimicrobials by Buul *et al*. | 34 (63.0) | 7 (13.0) | 13 (24.0) |

OMI, outcome measurement instrument
